# Equity and efficiency of primary health care resource allocation in mainland China

**DOI:** 10.1186/s12939-018-0851-8

**Published:** 2018-09-12

**Authors:** Yue Zhang, Qian Wang, Tian Jiang, Jian Wang

**Affiliations:** 1 School of Health Care Management, Shandong University, 44 Culture Road, Li Xia District, Jinan, 250012 Shandong Province China; 2 NHC Key Laboratory of Health Economics and Policy Research (Shandong University), Jinan, 250012 China; 3 School of Foreign Languages and Literature, Shandong University, 5 Hongjialou, Li Cheng District, Jinan, 250100 Shandong Province China

**Keywords:** Primary health care, Equity, Efficiency, Productivity

## Abstract

**Background:**

China had proposed the unification of equity and efficiency since the launch of the new round of health system reform in 2009. And the central government gave priority to the development of primary health care (PHC) whilst ensuring its availability and improving its efficiency. This study aimed to evaluate the changes of equity and efficiency in PHC resource allocation (PHCRA) and explored ways to improve the current situation.

**Methods:**

The data of this study came from the China Health Statistical Yearbook (2013–2017) and China Statistical Yearbook (2017). Three and five indicators were used to measure equity and efficiency, respectively. The Lorenz curve, Gini coefficient (*G*), Theil index (*T*) and health resource density index (HRDI) were used to assess equity in demographic and geographical dimensions. Data envelopment analysis (DEA) and the Malmquist productivity index (MPI) were chosen to measure the efficiency and productivity of PHCRA.

**Results:**

From 2012 to 2016, the total amount of PHCR had increased year by year. The *Gs* by population size were below 0.2 and that by geographical area were between 0.6 and 0.7. *T* had the same trend with *G*, and intra-regional contribution rates were higher than inter-regional contribution rates, which were all beyond 60%. From 2012 to 2016, the numbers of provinces that achieved an effective DEA were 4, 3, 4, 5 and 5, respectively. The mean of the total factor productivity index was 0.994.

**Conclusion:**

The equity of PHCRA in terms of population size is superior in the geographical area. Intra-regional differences are the main source of inequality. The eastern region has the highest density of PHCR, whereas the western region has the lowest. In addition, PHC institutions in more than 80% of the provinces are inefficient, and the productivity of the institutions decline by 0.6% from 2012 to 2016 because of technological retrogression.

## Background

The primary health care (PHC) system is the key component of almost every health system in the world [1]. China had always attached considerable importance to PHC and established a good PHC system as a model before 1978, which was highly praised by the World Health Organization. Subsequently, a market-oriented reform was implemented in the health sector. The efficient three-tier health-care delivery system and the Cooperative Medical System nearly collapsed [2, 3]. Fortunately, the Chinese government launched a new round of health care reform with a primary goal of rebuilding an effective PHC system [4]. The government had invested an additional $127 billion to enhance the infrastructures of PHC institutions, particularly those in rural areas [5]. These measures aimed at ensuring the commonweal of PHC and providing for all as a public product.

The reform had five key tasks [6] in 2009, one of the tasks was to build a relatively complete PHC system in 3 years (2009–2011). The Deepening Health Care Reform on March 14, 2012 proposed that we should consolidate and improve the service capabilities of PHC institutions. One of the goal of this reform is that the PHC will be more equitable and accessible, and efficiency of PHC institutions will be significantly improved by 2015. Specifically, the number of beds per thousand people will be 1.2 and the number of health workers per thousand people will be 3.5 in PHC institutions by 2020 (<Outline of the national plan for medical and health service system (2015–2020) > issued by the General Office of the State Council on March 6, 2015). Since 2009, the situation of PHC system and the equity and efficiency of the PHCRA were hot topics. In fact, equity and efficiency are always important goals pursued by health policy makers and health system [7]. And, some scholars had been devoted to research the equity and efficiency of health resource allocation. However, most of the studies were aimed at a certain area or one aspect and were based on data from early national yearbooks [8–10]. For instance, Zhang et al. [11] conducted a comparative study on inequality in the distribution of health resources and health services between hospitals and primary care institutions in China. Xu et al. [1] analyzed trends in the distribution of PHC professionals in Jiangsu Province of eastern China. In these studies, the methods that scholars mainly used to measure equity were Gini coefficient, Theil index, Concentration index, Lorenz curve and so on [12]. Lorenz curve could put a vivid reflection of the equity in resources allocation when combine with Gini coefficients, Theil index could reflect the contribution rate within the group and between groups when measuring the main factors causing the disparities [13]. Besides, Cheng et al. [4] used bootstrapping data envelopment analysis to assess the efficiency and productivity of rural township hospitals in China. Giuffrida researched productivity and efficiency changes in primary care based on a Malmquist index approach [14]. Indeed, DEA and MPI were extensively used to assess efficiency and productivity of decision-making units (DMUs) [15]. DEA is a method of performance evaluation that includes mathematical planning models to evaluate the relative effectiveness of the department or unit using multiple input and output indicators, and MPI is used in production analysis through the calculation of the ratio between the distance function to the productivity index [16]. Based on these successful practices of these methods in equity and efficiency measurement, this study adopted Lorenz curve, Gini coefficient and Theil index to measure equity, and DEA and MPI to measure efficiency and productivity. However, relatively few studies focused on nationwide PHCRA based on the latest data. And, those previous studies couldn’t represent the latest situation and if they only focused on some areas, some institutions or one aspect, they couldn’t represent the entire situation of China. Meanwhile, the nationwide study was easy to compare differences in provincial level and regions, then to develop corresponding plans to solve these problems.

The Deepening Health Care Reform from 2012 to 2015 had finished, the effects of this reform for PHCR need to be assessed in time. This study aimed to evaluate the equity, efficiency and productivity of PHCRA in this reform with the latest and nationwide data, analyse the causes of deficiencies, explore measures to solve the problems and provide references for policy makers in sustainable reform and other scholars.

## Methods

### Data sources

Data were extracted from the China Health Statistical Yearbook (2013–2017) and China Statistical Yearbook (2017), which covered 31 provinces, autonomous regions and municipalities. A series of time data (2012–2016) were used to analyse the trends of equity, efficiency and productivity and cross-sectional data (2016) were used to illustrate the variation of DMUs.

### Setting

On the basis of the geographical position, the economic development level and the China Health Statistical Yearbook, 31 provinces, autonomous regions and municipalities of mainland China were divided into three groups: eastern, central and western regions. The eastern region included Beijing, Tianjin, Hebei, Liaoning, Shanghai, Jiangsu, Zhejiang, Fujian, Shandong, Guangdong and Hainan (11 provinces and municipalities). The central region included Shanxi, Jilin, Heilongjiang, Anhui, Jiangxi, Henan, Hubei and Hunan (eight provinces). The western region included Inner Mongolia, Chongqing, Guangxi, Sichuan, Guizhou, Yunnan, Tibet, Shaanxi, Gansu, Qinghai, Ningxia and Xinjiang (12 provinces, autonomous regions and municipalities). PHC institutions included community health service centres (stations), street hospitals, township hospitals, village clinics, outpatient clinics and infirmaries.

### Indicators and measuring tools

Given the requirements of representation, availability, stability, independence [16] and consistency of previous studies [17–20], labour and capital were considered important input variables in the delivery of health services. The number of institutions and beds represents the capital, and health workers represents the human resources. The health workers include physicians, nurses, other clinical staff, administrative staff, and other nonclinical staff [21]. They were chosen as input indicators to measure equity. The average number of visits and the annual hospitalization rate as output indicators were combined with the input indicators to measure efficiency and productivity.

With regard to the Lorenz curve, the x-axis represents the cumulative percentage of population or geography, the y-axis shows the cumulative percentage of the PHCR (institutions, beds and health workers) and the diagonal line means absolute equity. The larger the distance from the absolute equality curve, the more the inequity [12, 22]. In this study, formula (1) [23] was used to calculate the value of the Gini coefficient (*G*). *G* ranges from 0 to 1; the closer the value to 0, the better the fairness; the closer the value to 1, the lesser the equity. Generally, *G* < 0.2 indicates absolute equality; 0.2–0.3, relative equality; 0.3–0.4, proper equality; 0.4–0.5, relative inequality; and above 0.5, severe inequality [24, 25].

1 $$ G=1-\sum \limits_{i=0}^{k-1}\left({Y}_{i+1}+{Y}_i\right)\left({X}_{i+1}-{X}_i\right) $$

*Y*_i_: cumulative percentage of the PHCR (institutions, beds and health workers) in the *ith* district.

*X*_*i*_: cumulative percentage of population or geography in the *ith* district.

*k*: total number of districts.

*G* can only describe the degree of equity, whereas *T* can be used to analyse the source of inequity. Inequity can be decomposed into intra- and inter-regions [26, 27]. However, *T* is a relative indicator, and no universal assessment standard of inequality levels is available [23]. Generally, the smaller the *T*, the greater the equity. *T* is calculated as follows:

2 $$ T=\sum \limits_{i=1}^n{P}_i\log \frac{P_i}{Y_i} $$

*P*_*i*_: proportion of every province’s population accounting for the overall China population.

*Y*_*i*_: proportion of PHCR owned by every province accounting for the total number of PHCR nationwide.

In formula (2), *T* can be divided into *T*_int *ra*_and *T*_int *er*_, and the calculation of *T*_int *ra*_ and *T*_int *er*_is as follows:

$$ T={T}_{\operatorname{int}\  ra}+{T}_{\operatorname{int}\  er}, $$

3 $$ {T}_{\operatorname{int}\  er}=\sum \limits_{g=1}^k{P}_g{T}_g, $$

4 $$ {T}_{\operatorname{int}\  er}=\sum \limits_{g=1}^k{P}_g\ln \frac{P_g}{Y_g}. $$

*T*_*g*_: *T* of the three groups (eastern, central and western regions).

*P*_*g*_: proportion of the three groups’ (eastern, central and western regions) population accounting for the overall population of China.

*Y*_*g*_: proportion of PHCR owned by the three groups (eastern, central and western regions) accounting for the total number of PHCR nationwide.

The contribution rate of intra- and inter-region can be calculated by dividing *T,* as $$ \raisebox{1ex}{${T}_{\operatorname{int} ra}$}\!\left/ \!\raisebox{-1ex}{$T$}\right. $$ and $$ \raisebox{1ex}{${T}_{\operatorname{int} er}$}\!\left/ \!\raisebox{-1ex}{$T$}\right. $$ [19].

Moreover, the HRDI was combined with the data map to show the differences in PHCRA in 31 provinces, autonomous regions and municipalities. The HRDI can mediate the influence of population and geographical factors to avoid bias caused by a single aspect of population or geographical area. The value of HRDI equaled the geometric mean of PHCR per thousand people and per square kilometre. Furthermore, the data map was drawn with a macro.

In view of the social welfare of PHC services and small demand elasticity, an input-oriented DEA was chosen, which was consistent with the study of Pelone et al. [28]. After comprehensively considering the actual situation, imperfect competition, government regulations and finance constraints, the PHC institutions always run at a suboptimal scale [29]. Thus, we preferred to select the BCC (developed by Banker, Charnes and Cooper) model under the assumption of variable returns to scale. In this model, technical efficiency (TE) can be decomposed into a product with pure technical efficiency (PTE) and scale efficiency (SE): TE = PTE * SE. However, DEA only measures relative efficiency for a period of time, but MPI can measure dynamic changes of productivity from time *t* to time *t* + 1 [30]. MPI is also called total factor productivity changes (TFPC), which can be divided into technical efficiency changes (TECs) and technological changes (TCs). TEC can also be decomposed into pure technical efficiency changes (PTECs) and scale efficiency changes (SECs) [31], that is, TFPC = TEC * TC; TEC = PTEC * SEC. TE is the production efficiency of the DMU based on a certain input factor, PTE indicates that managers’ hard work, personnel’s efforts and the correct combination of production factors have led to increased productivity [32]; SE reflects the different stages of the DMU’s economies of scale changes, TC mainly reflects the production of technological progress on the impact of changes in productivity [16]. A TE, PTE and SE value of one signifies efficiency; a TFPC, TEC, TC, PTEC and SEC value of more than one means improvement. All of the values were calculated by DEAP V.2.1 software [33]. The number of DMUs should be more than or equal to three times the sum of the numbers of indicators of inputs and outputs [34]; hence, the 31 provinces, autonomous regions and municipalities were chosen as DMUs.

## Results

### The equity of PHCRA

From 2012 to 2016, the amount of PHCR has increased. The average annual growth rates of institutions, beds and health workers are 0.379%, 2.151% and 1.739%, respectively. The PHCRA in terms of per thousand persons and per square kilometer are also increasing, except institution allocation according to the population (see Table 1).

**Table 1:** PHCRA from 2012 to 2016

Year	Institutions			Beds			Health workers		
	/1000 persons	/km^2^	Total	/1000 persons	/km^2^	Total	/1000 persons	/km^2^	Total
2012	0.6771	0.0950	912,620	0.9825	0.1378	1,324,270	2.5500	0.3577	3,437,172
2013	0.6755	0.0952	915,368	0.9961	0.1405	1,349,908	2.5932	0.3657	3,514,193
2014	0.6733	0.0955	917,335	1.0138	0.1437	1,381,197	2.5959	0.3680	3,536,754
2015	0.6717	0.0958	920,770	1.0313	0.1471	1,413,842	2.6284	0.3749	3,603,162
2016	0.6715	0.0964	926,518	1.0450	0.1500	1,441,940	2.6688	0.3832	3,682,561

**Fig. 1:**
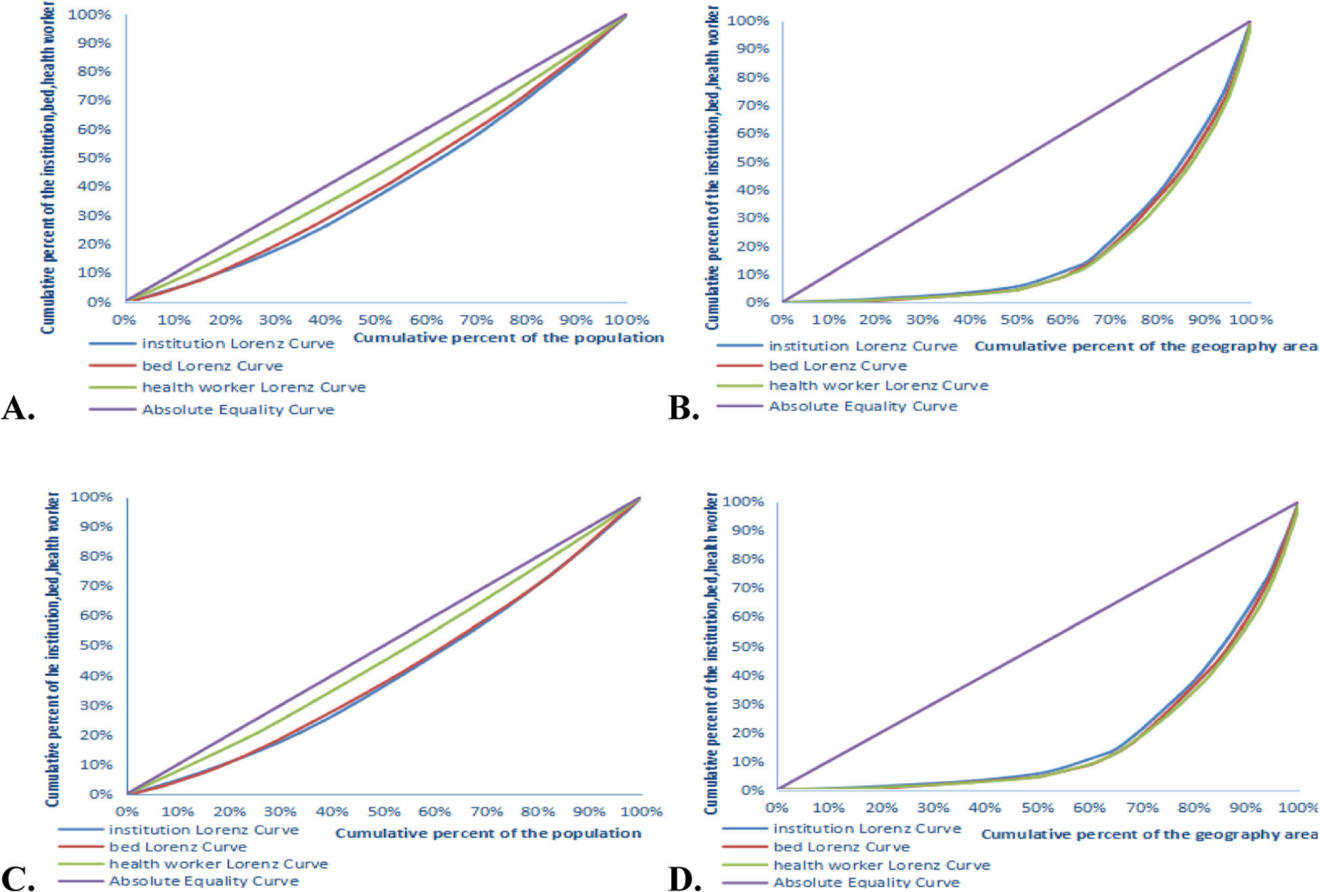
The Lorenz curves of PHCRA in 2012 and 2016. **a** and **b** denote the Lorenz curves of PHCRA in 2012, (**c**) and (**d**) denote the Lorenz curves of PHCRA in 2016. **a** and **c** are the demographic dimension; (**b**) and (**d**) are the geographical dimension

Figure 1 presents the Lorenz curves based on demographic and geographical dimensions. In principle, two figures were found every year. One figure was in the demographic dimension, and the other was in the geographical dimension. A total of 10 figures were found in 5 years (2012–2016). However, considering the limited space and presentation, we only show the four figures as follows:

As shown in the figures, the Lorenz curves in A and C were closer to the absolute equality curve. This finding indicated that the PHCRA in the demographic dimension was more equitable than that in the geographical dimension. The equity of health workers in the Lorenz curve was the closest to the absolute equality curve, and the equity of institutions in the Lorenz curve was the farthest in A and C. This finding affirmed that the equity of health workers was the best and that of the institutions was the worst in terms of the demographic dimension. According to B and D, the equity of institutions in Lorenz curve was the closest to the absolute equality curve, whereas that of the health workers was the farthest. This finding verified that the equity of institutions was the best and that of the health workers was the worst in terms of the geographical dimension. It was adverse to the demographic dimension. The figures in 2013, 2014 and 2015 had the same situation. Moreover, *G*s as shown in Table 2 were used to illustrate the trend of equity in PHCRA. When the PHCR was allocated by population, the *G*s were all less than 0.2, which means absolutely equitable. When the PHCR was allocated by geographical area, the *G*s were all larger than 0.6. It means severely inequitable. The *G* of institutions and health workers in population size from 2012 to 2016 presented a decreasing trend, which indicated that the trend of equity had improved. However, the *G* of beds increased, and its equity was worse. The worse trend of equity in beds was mainly caused by the differences of beds allocated in the east, middle and west, which were larger than those of the institutions and health workers. Specifically, the average numbers of institutions, beds and health workers per thousand people in east, middle and west were 0.5880, 0.6904, 0.7862, 0.8044, 1.1141, 1.2183, 2.5530, 2.6160 and 2.6802, respectively. In view of the geographical area, the equity in institutions showed a decreasing trend, which was mainly caused by the increase of institutions concentrated on east. The average numbers of institutions per km^2^ in east, middle and west were 0.3133, 0.1772, 0.0422. As for beds, the equity rose first and then fell, the turning point was in 2015. Because, the growth rate of 2015 in middle was larger than east and west, the values were 3.82%, 1.47%, 1.72%. And the equity of health workers presented a good trend except 2013.

**Table 2:** *G* of PHCRA by population and geographical area (2012–2016)

Year	Allocation by population			Allocation by geographical area		
	Institutions	Beds	Health workers	Institutions	Beds	Health workers
2012	0.1921	0.1659	0.0880	0.6153	0.6424	0.6544
2013	0.1913	0.1684	0.0902	0.6171	0.6423	0.6551
2014	0.1918	0.1743	0.0881	0.6170	0.6412	0.6533
2015	0.1913	0.1781	0.0816	0.6177	0.6430	0.6530
2016	0.1908	0.1804	0.0730	0.6176	0.6426	0.6531

The *T* of PHCRA in Table 3 showed the same tend with *G*, reflecting that its equity was similar to *G*. The further analysis of the sources validated that the inequality mostly came from intra-regional differences. The contribution rate of intra-region in institutions was approximately equal to 90%, and that of beds and health workers was approximately equal to 70% and more than 95%, respectively. Subsequently, we continued to decompose differences in intra-region (Table 4). Internal differences in the eastern region contributed the most to that in PHCRA. This finding means that the inequality of PHCRA mainly comes from the intra-eastern region. Differences in the intra-eastern region had the largest contribution to allocation of beds, approximately 70%, whereas that of institutions and health workers was approximately 60%. However, the differences in the intra-eastern and western regions decreased, whereas those in the central region were adverse. To clarify equity in PHCRA in the eastern, central and western regions, we calculated *T* of every region in Table 5. *T* of beds and health workers in the central region was the smallest and largest in the eastern region, respectively. This finding means that the allocation of beds and health workers in the central region is the most equitable and the worst in the eastern region, respectively. The allocation of institutions was best in the western region and worst in the eastern region. In addition, the equities of health workers in the eastern, central and western regions were all the best, and the institutions were all the worst.

**Table 3:** *T* of PHCRA by year

Year	Theil index			Contribution rate of intra-region (%)		
	Institutions	Beds	Health workers	Institutions	Beds	Health workers
2012	0.0657	0.0545	0.0125	88.65	76.65	98.25
2013	0.0660	0.0574	0.0133	89.08	74.41	99.18
2014	0.0660	0.0615	0.0128	89.11	71.14	98.00
2015	0.0656	0.0635	0.0113	89.13	70.73	97.58
2016	0.0648	0.0642	0.0091	89.41	69.35	96.99

**Table 4:** Proportion of differences in contribution in the intra-east, middle and west

Year	Institutions (%)			Beds (%)			Health workers (%)		
	East	Middle	West	East	Middle	West	East	Middle	West
2012	60.84	24.36	14.81	74.54	9.63	15.84	56.84	16.53	26.63
2013	60.63	24.67	14.71	74.14	10.53	15.33	62.46	15.61	21.92
2014	60.48	24.38	15.14	71.03	13.39	15.58	59.22	18.72	22.05
2015	59.88	25.15	14.96	67.95	16.66	15.39	55.68	23.30	21.02
2016	59.58	25.58	14.83	65.57	19.07	15.36	51.60	28.96	19.43

**Table 5:** *T* of PHCRA in the east, middle and west

Year	Institutions			Beds			Health workers		
	East	Middle	West	East	Middle	West	East	Middle	West
2012	0.0855	0.0450	0.0319	0.0752	0.0128	0.0245	0.0168	0.0064	0.0121
2013	0.0859	0.0460	0.0320	0.0764	0.0143	0.0242	0.0199	0.0065	0.0107
2014	0.0857	0.0456	0.0329	0.0749	0.0186	0.0252	0.0179	0.0075	0.0103
2015	0.0844	0.0469	0.0323	0.0735	0.0238	0.0255	0.0148	0.0082	0.0085
2016	0.0831	0.0473	0.0317	0.0703	0.0271	0.0252	0.0110	0.0082	0.0064

**Fig. 2:**
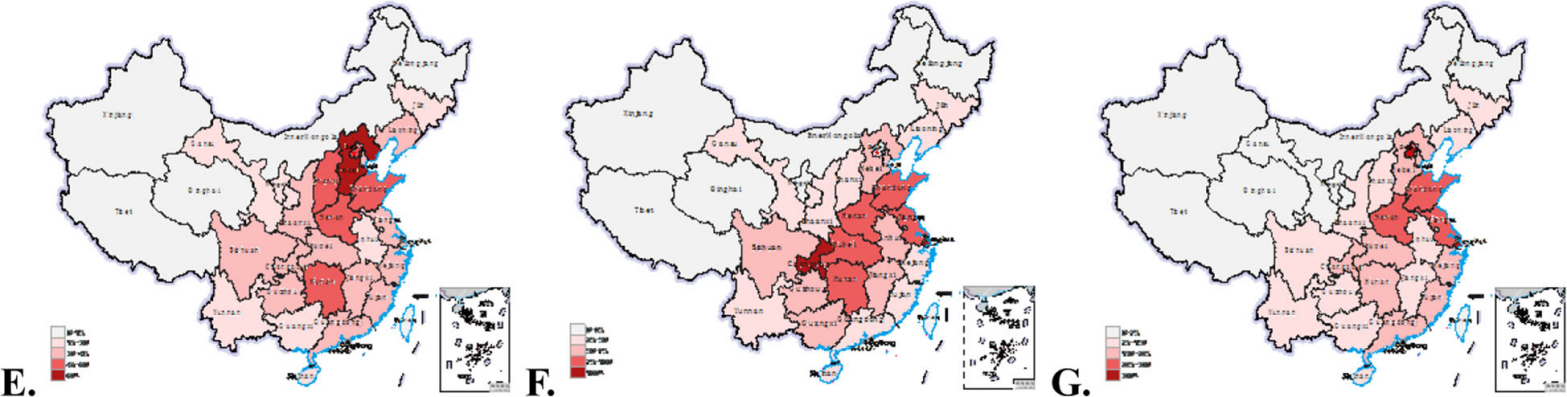
The HRDI (%) of PHCRA in 2016. (**e**, **f** and **g)** represent the HRDI (%) of institutions, beds and health workers allocation in 2016, respectively

Table 6 exhibits the HRDI of PHCRA. The HRDI in the eastern, central, western and national regions has been increasing in recent years. This finding means that the equity of PHCRA has been gradually improving. The equity of health workers was better than that of beds and institutions. The HRDI of PHCRA in the eastern and central regions was larger than that of the nationwide region, and the largest value was found in all eastern regions. Correspondingly, equity was the best in the eastern region and the worst in the western region. Meanwhile, the HRDI was combined with the data map to vividly show differences in 31 provinces, autonomous regions and municipalities in 2016 (Fig. 2). As shown in these figures, the equity of PHCRA from northwest to southeast had a trend of growth. In 2016, the largest value of HRDI for institutions, beds and health workers in Hebei province was seven times more than the smallest value in Xinjiang, and that of Shanghai was 25 times more than the smallest in Tibet and 18 times more than the smallest in Qinghai. Thus, relatively large differences were found among the provinces.

**Table 6:** HRDI of PHCRA by area and year

Year	Institutions				Beds				Health workers			
	East	Middle	West	Nation	East	Middle	West	Nation	East	Middle	West	Nation
2012	0.4270	0.3506	0.1822	0.2536	0.5834	0.5372	0.2668	0.3679	1.8040	1.3125	0.5952	0.9550
2013	0.4286	0.3501	0.1819	0.2536	0.5854	0.5430	0.2768	0.3741	1.8561	1.3174	0.6099	0.9738
2014	0.4291	0.3488	0.1820	0.2535	0.5827	0.5632	0.2847	0.3817	1.8488	1.3217	0.6195	0.9774
2015	0.4292	0.3495	0.1820	0.2537	0.5895	0.5833	0.2885	0.3895	1.8821	1.3316	0.6327	0.9927
2016	0.4320	0.3499	0.1821	0.2544	0.5947	0.5958	0.2941	0.3960	1.9274	1.3438	0.6464	1.0113

In recent years, with the acceleration of urbanization, differences in urban and rural regions have attracted much attention. These differences reflect in PHCRA, which are inequities between cities and countrysides. They had been showed in Table 7. More and more rural people were transferred to urban areas every year, a lot of health workers were attracted to urban institutions correspondingly. But, the growth rate of urban beds couldn’t catch up with that of population. Although, the people in rural areas are decreasing, many beds and health workers are still allocated to countrysides.

**Table 7:** Differences in urban and rural areas

Year	Population (1000 people)		Institutions		Beds		Health workers	
	Urban	Rural	Urban	Rural	Urban	Rural	Urban	Rural
2012	711,820	642,220	121,132	791,488	158,712	1,165,558	684,900	2,752,272
2013	731,110	629,610	127,508	787,860	147,793	1,202,115	729,207	2,784,986
2014	749,160	618,660	132,269	785,066	149,047	1,232,150	757,613	2,779,140
2015	771,160	603,460	140,686	780,084	153,959	1,259,883	809,933	2,793,229
2016	792,980	589,730	147,745	778,773	155,769	1,286,171	869,712	2,812,849
AAGR	2.74%	−2.11%	5.09%	−0.40%	−0.47%	2.49%	6.15%	0.55%

*AAGR* average annual growth rate

### The efficiency in PHC institutions

Table 8 presents an increasing trend of input indicators, but outputs do not have an evident increasing trend. Moreover, the annual hospitalization rate had declined from 2012 to 2016. Although TE and SE had increased, as shown in Table 9, the efficiency value was very low. PTE has a decreasing trend in recent years. Thus, the decline of PTE limited the improvement of TE. And, the number of the provinces that achieved a comprehensive efficiency was respectively 4, 3, 4, 5 and 5 from 2012 to 2016. Approximately 20 provinces had decreasing returns to scale each year. The data from 2016 (Table 10) was used to illustrate the variation of inputs and outputs needed to be adjusted in inefficient provinces. On the basis of the production frontier, 14 provinces only need to reduce their inputs, and 6 provinces need not only to reduce their inputs but also to improve their outputs.

**Table 8:** Descriptive statistics of inputs and outputs by year

Year	Items	Input			Output	
		I_1_	I_2_	I_3_	O_1_	O_2_
2012	Mean	29,439	42,718	110,877	2.789	2.789%
	Maxi	77,177	125,877	325,600	4.268	6.439%
	Mini	3904	2583	12,405	1.534	0.174%
2013	Mean	29,528	43,545	113,361	2.921	2.814%
	Maxi	75,178	125,964	348,424	4.506	6.592%
	Mini	3898	3087	12,578	1.584	0.161%
2014	Mean	29,591	44,555	114,089	2.937	2.637%
	Maxi	76,110	128,645	337,331	4.461	6.252%
	Mini	3918	3052	13,035	1.486	0.093%
2015	Mean	29,702	45,608	116,231	2.907	2.556%
	Maxi	76,214	130,741	331,438	4.777	6.066%
	Mini	3981	3198	13,366	1.327	0.092%
2016	Mean	29,888	46,514	118,792	2.909	2.614%
	Maxi	76,619	132,023	321,582	5.030	6.135%
	Mini	3968	3218	14,097	1.339	0.138%

**I**_**1**_**:** institutions; **I**_**2**_**:** beds; **I**_**3**_**:** health workers; **O**_**1**_**:** average number of visits; **O**_**2**_**:** annual hospitalization rate

**Table 9:** TE and SE of PHC institutions by year

Year	TE			PTE			SE		
	Mean	Maxi	Mini	Mean	Maxi	Mini	Mean	Maxi	Mini
2012	0.384	1.000	0.110	0.658	1.000	0.129	0.619	1.000	0.124
2013	0.398	1.000	0.113	0.648	1.000	0.132	0.633	1.000	0.115
2014	0.431	1.000	0.120	0.675	1.000	0.140	0.658	1.000	0.120
2015	0.472	1.000	0.128	0.654	1.000	0.144	0.726	1.000	0.227
2016	0.461	1.000	0.129	0.642	1.000	0.144	0.719	1.000	0.218

*TE* overall technical efficiency, *PTE* pure technical efficiency, *SE* scale efficiency = TE/PTE

**Table 10:** Variation of inputs and outputs needed to be adjusted in 2016

Provinces	Input			Output	
	I_1_	I_2_	I_3_	O_1_	O_2_
Beijing	0	0	0	0	0
Tianjin	−817	− 2298	− 9322	0	0.107%
Hebei	−59,830	−46,516	− 108,259	0	0
Liaoning	−28,932	−32,453	−82,295	0.128	0
Shanghai	0	0	0	0	0
Jiangsu	−18,597	−49,560	−147,109	0	0
Zhejiang	0	0	0	0	0
Fujian	−17,831	−17,603	−67,998	0	0
Shandong	−46,659	−75,750	− 189,679	0	0
Guangdong	−31,676	−45,164	−165,914	0	0
Hainan	0	0	0	0	0
Shanxi	−35,335	−32,821	−88,734	0.245	0
Jilin	−15,621	−17,678	−56,210	0.600	0.010%
Heilongjiang	−12,316	−24,737	−63,449	0.602	0
Anhui	−13,356	−40,915	−95,909	0	0
Jiangxi	−18,969	−13,284	−32,094	0	0
Henan	−39,405	−66,145	−144,695	0	0
Hubei	0	0	0	0	0
Hunan	−39,743	−59,361	−98,173	0.271	0
Inner Mongolia	−17,227	−20,120	−53,542	0	0
Chongqing	0	0	0	0	0
Guangxi	−12,346	−16,799	−53,450	0	0
Sichuan	0	0	0	0	0
Guizhou	−12,524	−20,647	−49,527	0	0
Yunnan	−11,942	−27,349	−57,506	0	0
Tibet	0	0	0	0	0
Shaanxi	−26,240	−24,880	−81,213	0	0
Gansu	−13,362	− 3990	− 9899	0	0
Qinghai	0	0	0	0	0
Ningxia	0	0	0	0	0
Xinjiang	0	0	0	0	0

### The productivity of PHC institutions

The MPI of annual means was used to analyse the productivity changes from 2012 to 2016 (Table 11). The geometric mean of TFPC was 0.994, which indicated that the productivity of PHC institutions in provinces had decreased by 0.6% from 2012 to 2016. Further analysis on the cause of the decline mainly showed that the TC had decreased by 6.2%. TEC increased by 6% because PTEC and SEC increased by 0.3% and 5.7%. Table 12 shows the MPI of provinces from 2012 to 2016. The TFPC of 17 provinces was more than one, which had improved productivity. However, 14 provinces still sank into deterioration in productivity. These provinces had experienced negative productivity changes from 2013 to 2015, but they went through a positive productivity from 2015 to 2016, which was why TC highly improved.

**Table 11:** MPI summary of annual means and frequency distribution by year

Year	TEC	TC	PETC	SEC	TFPC
2012–2013	1.047	0.973	0.996	1.051	1.018
2013–2014	1.106	0.878	1.058	1.045	0.971
2014–2015	1.117	0.871	0.976	1.145	0.973
2015–2016	0.976	1.039	0.983	0.992	1.014
Mean	1.060	0.938	1.003	1.057	0.994
Frequency distribution (2012–2013)					
> 1	22	5	14	13	19
1	3	1	10	3	0
< 1	6	25	7	15	12
Frequency distribution (2013–2014)					
> 1	26	6	16	20	10
1	3	0	10	3	0
< 1	2	25	5	8	21
Frequency distribution (2014–2015)					
> 1	25	1	12	24	10
1	4	0	10	4	0
< 1	2	30	9	3	21
Frequency distribution (2015–2016)					
> 1	11	26	9	10	18
1	4	0	10	4	0
< 1	16	5	12	17	13

*TECs* technical efficiency changes, TCs: technological changes, *PTECs* pure technical efficiency changes, *SECs* scale efficiency changes, *TFPCs* total factor productivity changes. A score > 1 indicates growth; a score of 1 signifies stagnation; a score < 1 indicates decline or deterioration

**Table 12:** MPI summary of means by province

Provinces	TEC	TC	PETC	SEC	TFPC
Beijing	1.055	0.988	1.000	1.055	1.042
Tianjin	0.968	1.011	0.964	1.004	0.978
Hebei	1.039	0.965	0.962	1.081	1.003
Liaoning	1.038	0.955	1.031	1.007	0.992
Shanghai	1.000	1.014	1.000	1.000	1.014
Jiangsu	1.103	0.945	1.129	0.977	1.042
Zhejiang	1.029	1.011	1.000	1.029	1.041
Fujian	1.031	0.918	0.945	1.090	0.946
Shandong	1.015	0.950	0.800	1.269	0.964
Guangdong	1.033	0.975	0.993	1.040	1.007
Hainan	1.032	0.996	1.007	1.025	1.029
Shanxi	1.025	0.957	1.028	0.997	0.981
Jilin	0.977	0.981	0.979	0.998	0.958
Heilongjiang	1.180	0.904	1.093	1.079	1.066
Anhui	1.078	0.909	1.060	1.017	0.979
Jiangxi	1.099	0.878	0.989	1.112	0.966
Henan	1.070	0.935	0.940	1.138	1.000
Hubei	1.159	0.901	1.016	1.141	1.044
Hunan	1.171	0.877	1.072	1.093	1.027
Inner Mongolia	1.051	0.955	1.045	1.005	1.003
Chongqing	1.118	0.878	1.000	1.118	0.981
Guangxi	1.151	0.878	0.924	1.245	1.010
Sichuan	1.096	0.878	1.000	1.096	0.962
Guizhou	0.995	0.878	0.852	1.167	0.873
Yunnan	1.108	0.903	1.050	1.055	1.000
Tibet	1.000	0.987	1.000	1.000	0.987
Shaanxi	1.063	0.945	1.082	0.982	1.004
Gansu	1.051	0.960	1.049	1.002	1.009
Qinghai	1.000	0.907	1.000	1.000	0.907
Ningxia	1.000	0.985	1.000	1.000	0.985
Xinjiang	1.173	0.878	1.153	1.017	1.029
Mean	1.060	0.938	1.003	1.057	0.994

## Discussion

After a long period of development, China has established the medical and health service system, including PHC institutions. This study comprehensively analyzed the equity and efficiency of PHCRA in recent years. The results corroborated that, except for the number of institutions per capita, the total number of institutions, beds and health workers and the number of per capita and per km^2^ presented a steadily increasing trend. However, the speed of decrease in institutions per thousand capita slowed down, which was mainly due to the fact that the state, in recent years, had encouraged social capital to enter the medical market to improve the equity and efficiency of resource allocation through competition with public institutions. In terms of PHC institutions, nonpublic institutions occupied 46% in 2016. In 2016, the speed of growth in bed allocation slowed down. And, the differences in urban and rural areas showed that the number of beds per thousand people in urban regions was decreasing, which had limited the speed of increase in beds. If it does not improve, then achieving 1.2 beds per thousand capita in the PHC institutions will be difficult by 2020, which is the goal proposed by China. Compared with institutions and beds, the speed of growth in health workers improved. But, with respect to the number of health workers per thousand people, which in rural areas is more than four times as urban areas. So, in order to achieve the goal of 3.5 health workers per thousand capita by 2020, numerous health workers should be allocated to PHC institutions in urban areas gradually.

The Chinese government has issued a number of documents to optimize the allocation of health resources. However, most of these documents were based on population allocation. Correspondingly, the equity of resource allocation by population size was much better than that by geographical area. Many scholars have obtained the same conclusion [35, 36]. *G* of population size (0.07–0.19) was far less than *G* of geographical area (0.62–0.66). Indeed, when Zhang et al. [11] and Yang et al. [37] used *G* to measure equity of PHCRA by geographical area in China, the *Gs* were over 0.7 and between 0.6 and 0.7 respectively. Specifically, the equity of PHCRA by population size is health workers>beds>institutions. The equity of geography is institutions>beds> health workers. Zhang [11] and Yang [37] had also verified this result. The equity of *T* and *G* is the same as regards population size. Moreover, equity in the eastern, central and western regions were health workers>beds>institutions, and the differences in the intra-east mainly caused the inequity. The finding is the same as that of the study of Liu et al. and Gong et al. [13, 38]. By taking the number of institutions per thousand capita in 2016 as an example, seven provinces in eastern region (7/11) wre lower than their average, and the maximum value of Hubei was six times that of Shanghai. As for central and western regions, the proportions were 3/8, 6/12, and Shanxi was three times that of Anhui, Tibet was four times that of Yunnan respectively. The instance presented above could roughly explain the big differences in the intra-east. And, the differences contributed by the intra-east and west decreased; but, those of the central region increased. This finding means that the eastern region, with its economic and geographical advantages, is starting to improve its poor PHCRA [13]. The equity of western region had considerably improved because of inclined policies and aids [21]. However, the central region presented a relatively reduced development due to shortage of priority and positivity. When HRDI was used to assess the comprehensive equity, we found the following trend: eastern region>central region>western region. This reason may be the inadequate financial capacity and similar health resource allocation standards that cannot be followed [13]. The severe inequity in geographical area is indisputable, but existing policy documents are still based on population and administrative divisions to allocate health resources. However, a reasonable method is that the demographic and geographical distributions and all influencing factors should be considered when making health allocation plans [39]. Therefore, the central government should convert implementations of supply side oriented resource allocation to demand side oriented resource allocation and continue to increase inputs to central and western regions [40], narrow the regional disparities through financial transfer coordination [11], strengthen supervision and ensure that government investments can reach less developed and remote areas in a timely manner. Besides, more resources should be put into PHC institutions in urban areas gradually, because of increasing demand for services brought about by urbanization.

The results of the analysis on efficiency and productivity verified that PHC institutions had experienced significant technical inefficiency from 2012 to 2016. Although the government had attached importance to the PHC services, less than 20% of provinces achieved technical efficiency every year. The scores of SE (0.62–0.73) and PTE (0.64–0.68) were relatively bigger than those of TE (0.38–0.47). This finding means that these provinces only attained less than 50% of efficiency, and PTE, SE have a lot of room for improvement. Compared with similar studies, the mean of TE in this study is lower than that in the Nouna health district is 0.862 [41] and 0.833 in the Spanish region of Extremadura [42], 0.620 in the Weifang Prefecture [43] and 0.515 in the Xiaogan Prefecture of China [4]. Further analysis of the mean of TE, PTE and SE in the eastern, central and western regions showed that west>east>middle. The same result had been found in the study of Ding et al. [21]. This finding seemed to be an interesting phenomenon. The eastern region had economic and technical advantages and it’s amount of resources was larger than the others. Thus, their efficiency should be high. However, the scale of institutions in the east is too large, and the PHC institutions in east are mostly decreasing back to scale. Meanwhile, a much larger number of input redundancies have not been fully utilized. The technical level in the west region is limited, but their inputs are relatively appropriate and more fully utilized. As one of the output indicators, annual hospitalization rate can explain the differences, which is 4.063% in the western region and 1.934% in the eastern region. And, a study conducted by Zhang et al. [11] pointed out that richer people were more likely to go to high level hospitals for outpatient care, while poorer people were more likely to go to PHC institutions for inpatient care. Indeed, the economic development level of west is worse than east, geographical area is larger, traffic is inconvenient, so western people are more likely to go to PHC institutions. To some extent, the inputs and outputs in the western region were valuable to refer. Moreover, the low efficiency in the central region is reflected in the redundant inputs and inadequate outputs that simultaneously exist. Indeed, comparing with west and east, the central region did not benefit from the preferential regional policies and had become the most vulnerable region [44]. Specifically, Shanghai, Hainan (east), Qinghai and Ningxia (west) had no adjustment in their inputs, outputs and scale with their current technical level. Beijing, Zhejiang (east), Hubei (central), Chongqing, Sichuan and Xinjiang (west) only need to shrink scale, whereas others should improve TE and adjust scale.

Low TE had changed positively from 2012 to 2015. The productivity of institutions had risen again in 2016 because TE decline was counteracted by technological progress. Li et al. [16] asserted that technological progress was helpful to the short-term development of PHC services. However, to achieve sustainable development, the improvements of TE should be paid considerable attention. As for TE, because it decomposes into PTE and SE, so we should try our best to improve PTE and SE. Therefore, modifying manager and employee relationships, correcting leadership, marking personnel’s comments and suggestions, promoting and encouraging innovation and creating a favorable working environment are factors that can be effective in improving TE [32]. Finally, the medical pattern will be optimal which is small diseases in PHC institutions, severe diseases in hospitals, rehabilitation back to the PHC institutions with hierarchical treatment through referral system. Indeed, the study of Yip et al. [45] had proven that the PHC institutions could improve their overall efficiency and productivity by providing medical care, disease prevention, health promotion and education, rehabilitation and birth control [1]. However, the redundant inputs of 2016 are evidently larger than those of other years. These redundancies implied that PHC services did not meet the needs of the participating subjects (PHC institutions, primary doctors and patients). Specifically, services delivered by PHC institutions were deemed poor quality, leading to patients’ distrust [11]. Consequently, patients bypassed PHC institutions and received treatment in high-level health-care facilities [46]. In addition, additional resources were concentrated on hospitals and the residents’ income had been increasing because of market power, promoting patients to seek high-quality services from hospitals [47]. Meanwhile, the implementation of zero-profit policy of medicine and the National Essential Medicines Scheme had resulted in the reduction of drug income in institutions and the corresponding reduction of staffs’ performance salary. At this time, government subsidies were difficult to be timely in place, resulting in the overall work enthusiasm frustrated [4]. In fact, the real reasons for the decline in TE are complex, but the analysis here only discovers one aspect, thus more studies are needed to do this complex analysis.

### Limitation

In this study, not only general methods were used to assess the equity and efficiency based on the latest data but also HRDI was combined with the data map to vividly show the differences in equity. However, some limitations also presented. Firstly, although the selection of indicators was consistent with that of previous studies, the results of different combinations of indicators were not measured. Moreover, some indicators, such as the utilization and turnover rate of beds, were not included in this study due to the unavailability of data. Secondly, when assessing the equity regarding the demographic allocation, only the resident population was considered the targeted population in this study, and this assessment of equity may influence the reflection of the real situation because of the presence of migrants [48]. Thirdly, bias adjustments of efficiency and productivity scores were not carried out due to the limitation of basic DEA approach [15]. Finally, the influences of environmental factors (e.g. New Cooperative Medical System reform) on the efficiency scores were not taken into account [4].

## Conclusion

This study provided an empirical research for the equity, efficiency and productivity of PHCRA based on authoritative data. The results corroborated that the equity of PHCRA had improved year by year and that the equity of population allocation was far better than the geographical area in China. After comprehensive consideration of population and geographical area factor, we confirmed that the eastern region had the largest resource density and best equity and the western region had contrary results. The results of the efficiency analysis affirmed that the TE and productivity of the institutions in each province were generally low. Further analysis of the invalid provinces elucidated that their scales were too large and had redundancies. The improvement of productivity relied solely on technological progress rather than on the improvement of internal management level and institutional innovation. The inputs of several resources had certainly caused the improvement of equity, but the improvement of TE had not kept up with the pace. In the future, internal management should be strengthen by setting performance goals, improving incentives and updating personnel quality. The existing resources should be fully and rationally applied, and the allocation of health resources in China should be revitalized through the flow of resources among the institutions.

## Abbreviations

DEA: data envelopment analysis
DMUs: decision-making units
*G*: Gini coefficient
HRDI: health resource density index
MPI: Malmquist productivity index
PHCRA: primary health care resource allocation
PTE: pure technical efficiency
PTECs: pure technical efficiency changes
SE: scale efficiency
SECs: scale efficiency changes
*T*: Theil index
TCs: technological changes
TE: technical efficiency
TECs: technical efficiency changes
TFPCs: total factor productivity changes

## References

[1] Xu K, Zhang K, Wang D (2014). Trend in distribution of primary health care professionals in Jiangsu province of eastern China. Int J Equity Health.

[2] Liu X, Xu L, Wang S (1996). Reforming China’s 50,000 township hospitals-effectiveness, challenges and opportunities. Health Policy.

[3] Wagstaff A, Lindelow M, Wang SY, et al. Reforming China's rural health system. Washington: World Bank. 2009. p. 1–248.

[4] Cheng Z, Cai M, Tao H, He Z (2016). Efficiency and productivity measurement of rural township hospitals in China: a bootstrapping data envelopment analysis. BMJ Open.

[5] Liu Q, Wang B, Kong Y (2011). China’s primary health-care reform. Lancet.

[6] Yip W, Hsiao W (2009). China’s health care reform: a tentative assessment. China Econ Rev.

[7] Zhou Z, Zhu L, Zhou Z (2014). The effects of China’s urban basic medical insurance schemes on the equity of health service utilization: evidence from Shaanxi Province. Int J Equity Health.

[8] Shen Y, Yan H, Reija K (2014). Equity in use of maternal health services in western rural China: a survey from Shaanxi province. BMC Health Serv Res.

[9] Liu Y, Jiang Y, Tang S (2015). Analysis of the equity of emergency medical services: a cross-sectional survey in Chongqing city. Int J Equity Health.

[10] Zhou Z, Gao J, Ashley F (2011). Measuring the equity of inpatient utilization in Chinese rural areas. BMC Health Serv Res.

[11] Zhang T, Xu Y, Ren J (2017). Inequality in the distribution of health resources and health services in China: hospitals versus primary care institutions. Int J Equity Health.

[12] Tao Y, Henry K, Zou Q (2014). Methods for measuring horizontal equity in health resource allocation: a comparative study. Health Econ Rev.

[13] Liu W, Liu Y, Twum P (2016). National equity of health resource allocation in China: data from 2009 to 2013. Int J Equity Health.

[14] Giuffrida A (1999). Productivity and efficiency changes in primary care: a Malmquist index approach. Health Care Manag Sci.

[15] Cheng Z, Tao H, Cai M (2015). Technical efficiency and productivity of Chinese county hospitals: an exploratory study in Henan province. China BMJ Open.

[16] Li NN, Wang CH, Ni H (2017). Efficiency and productivity of county-level public hospitals based on the data envelopment analysis model and Malmquist index in Anhui. China Chin Med J (Engl).

[17] Akbari Sari A, Rezaei S, Homaie Rad E (2015). Regional disparity in physical resources in the health sector in Iran: a comparison of two time periods. Iran J Public Health.

[18] Macinko J, Lima-Costa MF (2012). Horizontal equity in health care utilization in Brazil, 1998-2008. Int J Equity Health.

[19] Fang P, Dong S, Xiao J (2010). Regional inequality in health and its determinants: evidence from China. Health Policy.

[20] Sun J, Luo H (2017). Evaluation on equality and efficiency of health resources allocation and health services utilization in China. Int J Equity Health.

[21] Ding J, Hu X, Zhang X (2018). Equity and efficiency of medical service systems at the provincial level of China's mainland: a comparative study from 2009 to 2014. BMC Public Health.

[22] Theodorakis PN, Mantzavinis GD, Rrumbullaku L (2006). Measuring health inequalities in Albania: a focus on the distribution of general practitioners. Hum Resour Health.

[23] Brown MC (1994). Using Gini-style indexes to evaluate the spatial patterns of health practitioners-theoretical considerations and an application based on Alberta data. Soc Sci Med.

[24] Teng F, He J, Pan X (2011). Metric of carbon equity: carbon Gini index based on historical cumulative emission per capita. Adv Clim Chang Res.

[25] Zhou Y, Qin Y. Empirical analysis on income inequality of Chinese residents. New York: Springer. 2012; p. 1.

[26] Anand S (2010). Measuring health workforce inequalities: methods and application to China and India. Clin Chim Acta.

[27] Wang Y, Tu Q, Lai Q (2017). Fairness or not? Health resources allocation in Chongqing-based on Theil index. Open Journal of Social Sciences.

[28] Pelone F, Kringos DS, Romaniello A (2015). Primary care efficiency measurement using data envelopment analysis: a systematic review. J Med Syst.

[29] Coelli TJ, Rao DSP, O’Donnell CJ (2005). An introduction to efficiency and productivity analysis.

[30] Färe R, Grosskopf S, Lindgren B (1994). Productivity developments in Swedish hospitals: a Malmquist output index approach.

[31] Ying CN (2011). The productive efficiency of Chinese hospitals. China Econ Rev.

[32] Nabilou B, Yusefzadeh H, Rezapour A (2016). The productivity and its barriers in public hospitals: case study of ran. Med J Islam Repub Iran.

[33] Coelli T. A Guide to DEAP version 2.1: a data envelopment analysis (computer) program. 1996;1–49.

[34] Banker RD, Morey RC (1989). Incorporating value judgements in effciency analysis. Res Governmental Nonprofit Account.

[35] Jian J, Wang J, Ma X (2015). Equality of medical health resource allocation in China based on the Gini coefficient method. Iran J Public Health.

[36] He MH, Wang L, He J, Wei Y (2013). Status and equity of health services resources allocation in China. Chin Health Service Manage.

[37] Yang Z, Xiao H U, Chen R, et al. Research on the equity of the primary medical and health resource allocation in China. Chin Health Resour 2017, 20:106–109 (in Chinese).

[38] Gong XG, Hu SL (2005). Fairness of health resources allocation in China. Chin J Hosp Admin.

[39] Song P, Ren Z, Chang X (2016). Inequality of Paediatric workforce distribution in China. Int J Environ Res Public Health.

[40] Huang XP, Tang LX (2010). Region differences in hospital bed allocation in China. Chin J Health Policy.

[41] Marschall P, Flessa S (2011). Efficiency of primary care in rural Burkina Faso. A two-stage DEA analysis. Health Econ Rev.

[42] Cordero Ferrera JM, Cebada EC, Murillo Zamorano LR (2014). The effect of quality and socio-demographic variables on efficiency measures in primary health care. Eur J Health Econ.

[43] Audibert M, Mathonnat J, Pelissier A (2013). Health insurance reform and efficiency of township hospitals in rural China: an analysis from survey data. China Econ Rev.

[44] Wang LJ. To evaluate the traditional chinese medical service efficiency of the county traditional chinese medicine hospitals in Shangdong province. Chinese Health Economics. 2013;5:81–83. (in Chinese)

[45] Yip W, Hsiao W (2014). Harnessing the privatization of China’s fragmented health-care delivery. Lancet.

[46] World Bank (1997). Financing health care: issues and options for China.

[47] Zhao Y, Chen R, Wang B, Wu T (2014). General practice on-the-job training in Chinese urban community: a qualitative study on needs and challenges. PLoS One.

[48] Daraio C, Advanced Robust SL (2007). Nonparametric methods in efficiency analysis. J Econ Lit.

